# Some Like It Fat: Comparative Ultrastructure of the Embryo in Two Demosponges of the Genus *Mycale* (Order Poecilosclerida) from Antarctica and the Caribbean

**DOI:** 10.1371/journal.pone.0118805

**Published:** 2015-03-18

**Authors:** Ana Riesgo, Sergio Taboada, Laura Sánchez-Vila, Joan Solà, Andrea Bertran, Conxita Avila

**Affiliations:** 1 Department of Animal Biology, Faculty of Biology, Universitat de Barcelona, Barcelona, Spain; 2 Biodiversity Research Institute (IrBIO), Faculty of Biology, Universitat de Barcelona, Barcelona, Spain; Victoria University Wellington, NEW ZEALAND

## Abstract

During embryogenesis, organisms with lecithotrophic indirect development usually accumulate large quantities of energetic reserves in the form of yolk that are necessary for larval survival. Since all sponges have lecithotrophic development, yolk formation is an ineludible step of their embryogenesis. Sponge yolk platelets have a wide range of morphological forms, from entirely lipid or protein platelets to a combined platelet showing both lipids and proteins and even glycogen. So far, there are no comparative studies on the nature and content of yolk in congeneric species of sponges inhabiting contrasting environments, which could have putative effects on the larval adaptation to environmental conditions. Here, we have taken advantage of the worldwide distribution of the sponge genus *Mycale*, in order to compare the embryogenesis and yolk formation in two species inhabiting contrasting latitudinal areas: *M*. *acerata* from Antarctic waters and *M*. *laevis* from the Caribbean. We have compared their brooded embryos and larvae using scanning and transmission electron microscopy, and calculated their energetic signatures based on the nature of their yolk. While the general morphological feature of embryos and larvae of both species were very similar, the main difference resided in the yolk nature. The Antarctic species, *M*. *acerata*, showed exclusively lipid yolk, whereas the Caribbean species, *M*. *laevis*, showed combined platelets of lipids and proteins and less frequently protein yolk platelets. The larvae of *M*. *acerata* were estimated to possess a two-fold energetic signature compared to that of *M*. *laevis*, which may have important ecological implications for their survival and for maintaining large population densities in the cold waters of the Southern Ocean.

## Introduction

Embryogenesis in sponges is a highly diverse process that can occur in the water column in oviparous species or within the sponge body in viviparous species (see reviews by [1, 2, 3, 4]), where it has more frequently been described [4]. During sponge embryogenesis, several different morphogenetic movements often considered as a primitive form of gastrulation can occur, including delamination, invagination, and unipolar and multipolar egression (see [3] for a review). Besides morphogenetic movements, one of the most important processes during sponge embryogenesis is vitellogenesis, since all sponge larvae are lecithotrophic [5]. Even though vitellogenesis has its onset during oocyte maturation in sponges, much of the yolk is produced during embryogenesis [1, 2, 6, 7, 8].

There are many different mechanisms for yolk formation described in sponges: autosynthesis through the Golgi apparatus, heterosynthesis through nurse cells [1, 2, 8, 9], and more recently heterosynthesis using bacteria as primary material [10]. Yolk usually comprises several different cellular inclusions with either protein or lipid nature [6, 7]. In sponges yolk commonly presents a highly heterogeneous appearance, being both proteins and lipids compacted into the same platelet (e.g., [10, 11, 12, 13, 15, 14, 16]). However, some sponges show homogenous yolk of either protein or lipid nature (e.g., [9, 17, 18, 19, 20, 21]).

The occurrence of different yolk types among sponges shows no apparent phylogenetic affiliation, and it could well be related to the ecological niche occupied by the sponges, although this latter possibility has never been approached. Comparative studies of yolk in congeneric species might shed light into this issue. While in annelid polychaetes it has been shown that the yolk content in both nature and abundance can vary among congeneric species [22], in sponges such studies have never been performed. The different yolk types observed in polychaete eggs, for instance, have been related to the developmental mode of the species, being the protein yolk platelets in lecithotrophic species larger than those in planctotrophic species [22]. In addition, protein yolk appears to have a significantly higher energetic signature, while lipids appear as a rapid fuel for active cell division and differentiation [23]. Interestingly, the cost of protein synthesis in cold marine environments can be much higher when compared to that in warmer waters [24, 25], being up to 87% of the metabolic rate accounted for the energy costs of protein synthesis and other processes [25].

The genus *Mycale* is widely distributed in the planet, inhabiting all oceans, sometimes being the dominant species on some ecosystems [26, 27]. In Antarctic waters, *Mycale* (*Oxymycale*) *acerata* Kirkpatrick, 1907 is a common and abundant massive yellow sponge on the sublittoral rocky bottoms [27, 28, 29].It is a space-dominating sponge (reaching densities of 0.02 individuals/m^2^ and average height of 1.5 m) with high growth rates [27, 28, 30]. In Caribbean waters, *Mycale* (*Mycale*) *laevis* (Carter, 1882) is also a very common component of the benthic assemblage, growing sometimes in close association with scleractinian corals [31, 32], and exhibiting a great morphological variability, with four morphotypes quite conserved at the genetic level [33]. Poecilosclerid sponges, including the genus *Mycale*, are mostly hermaphroditic and viviparous [1, 2, 3, 34] and their embryonic development involves large production of yolk platelets and lipid droplets in some species (e.g., [35]). The hermaphroditic reproductive cycle of only two species of the genus *Mycale* has been investigated so far, including *Mycale* (*Aegogropila*) *contarenii* [36] from the Atlantic and Mediterranean [37, 38] and *Mycale* (*Carmia*) *micracanthoxea* Buizer & van Soest, 1977 from Atlantic waters [39]. The larval morphology, though, has been described for a number of species using light microscopy [39, 40, 41, 42, 43, 44, 45]. All species of *Mycale* possess large embryos and larvae (ranging from 600 to 800 μm in largest diameter), with oval to pear shape, strong pigmentation, and spicules [39, 40, 42, 43, 45, 46, 47]. However, the ultrastructure of their larva has been rarely investigated [37, 48, 49].

Here we provide the first comparative study of two species of the genus *Mycale*, *M*. (*Oxymycale*) *acerata* and *M*. (*Mycale*) *laevis* inhabiting radically different environments, the Southern Ocean and the Caribbean Sea, respectively. We studied the ultrastructure of embryos and larvae of both species using a comparative approach, focusing on species-specific adaptations in terms of yolk types and abundances, with possible relation to life in cold waters.

## Material and Methods

### Sample collection

**Fig 1:**
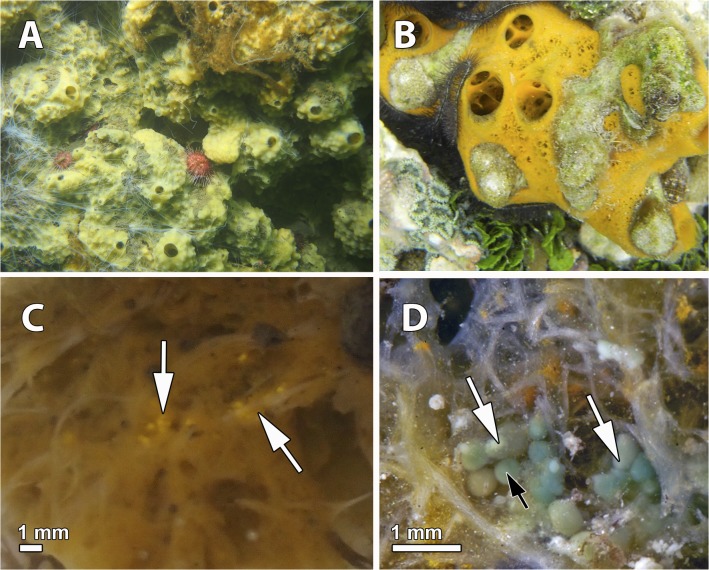
Live specimens of *Mycale* in the study areas. **A.** Massive specimens of living *Mycale acerata* in Deception Island, Antarctica. B. Adult specimen of *Mycale laevis* in Bocas del Toro, Panama. C. Embryos (arrows) brooded in the mesohyl of *M*. *acerata*. D. Embryos (white arrows) and ciliated larvae with a bare posterior pole (black arrow) within the mesohyl of *M*. *laevis*.

Samples of *Mycale* (*Oxymycale*) *acerata* (Fig. 1A) were collected by SCUBA diving on rocky outcrops at 15 m depth, Deception Island (62°59'31.20" S, 60°33'5.07" W, South Shetland Islands, Antarctica) in February 2^nd^-20^th^, 2013. Permission for collection of marine invertebrates was issued by the Spanish Ministry of Science and Innovation (CPE-EIA-2011-7). Out of the 5 specimens collected, only 2 harbored brooding embryos (n = 19, 30, Ntotal = 49). Samples from *Mycale* (*Mycale*) *laevis* (orange morph, phenotype massive) (Fig. 1B) were also collected by SCUBA diving on coral rock at 5–10 m depth, Bastimentos Island (9°20'44.93" N, 82°12'51.82" W, Bocas del Toro, Panama) in March 14^th^-17^th^, 2010. Permission for collection of marine invertebrates was issued by the Aquatic Resources Authorities of Panama (PO #212995). Out of the 10 individuals collected, only 3 of them had brooding embryos (n = 11, 9, 5, Ntotal = 25) and larvae (n = 22, 18, 29, Ntotal = 69) within their bodies.

### Light and electron microscopy

Samples were either preserved in 4% formalin for light microscopy or preserved in 2.5% glutaraldehyde in PBS for both scanning and transmission electron microscopy following protocols detailed in [21]. Formalin-preserved samples were later rinsed for 2 h in distilled water and dehydrated through an ascending series of ethanol (70%, 96%, 100%) and xylene. Then, samples were embedded in paraffin at 60°C overnight, and cut with a Microtome Micron HM325 to 5 μm. Staining was performed using Methylene blue and Hematoxilin-Eosin standard protocols. Samples for transmission electron microscopy preserved in 2.5% glutaraldehyde in PBS were then rinsed using a solution of 0.6M NaCl and PBS and fixed for 1h at 4°C in 1% osmium tetroxide-potassium ferrocyanide. Later, samples were rinsed in PBS and distilled water several times, dehydrated though a series of ethanol and propylene oxide, and embedded in Spurr resin for 3 days. Sections of resin blocks were performed at 64 nm using an ULTRACUT ultramicrotome, stained with lead citrate and uranyl acetate and observed with a JEOL 1010 electron microscope with a Gatan module for image digitalization at the Microscopy Unit at the Scientific and Technological Centers (CCiT), Universitat de Barcelona. Samples preserved in 2.5% glutaraldehyde in PBS for scanning electron microscopy were dehydrated through an increasing ethanol series and critically-point dried. After that, samples were coated with carbon, mounted in stabs, and observed with a JEOL 7100F Field Emission scanning electron microscope with a Gatan module for image digitalization at the Microscopy Unit of the CCiT, Universitat de Barcelona.

### Yolk/lipid ratio estimations

Blastomeres and larval cells were measured in 43 embryos of *Mycale acerata* and 17 embryos and 65 larvae of *M*. *laevis* using the software ImageJ 1.48 (http://imagej.nih.gov/ij/). Transmission electron micrographs from 6 embryos of *M*. *acerata* and 6 embryos and 4 larvae of *M*. *laevis* were used to estimate the protein/lipid ratios using the software ImageJ. The specific measurements obtained to be used as a proxy to estimate total volume of lipids and proteins in blastomeres and larval cells were as follows: average volume of embryos and larvae, number of blastomeres or larval cells containing yolk in the embryo/larva, average volume of embryo/larval cells, average number of heterogeneous platelet/homogeneous (lipid or protein) platelet per cell (measured in 10–15 cells per embryo/larvae, n = 90 in *M*. *acerata*, n = 150 in *M*. *laevis*), and average volume of heterogeneous platelet/homogeneous (lipid or protein) platelet (measured in 15 cells per embryo/larvae, n = 90 in *M*. *acerata*, n = 150 in *M*. *laevis*).

The estimation of energetic signatures was performed on total volume of lipids and proteins in blastomere and larval cells using the Energy Conversion Factors (ECF, for lipids is 7.9 kcal/g and for proteins is 4.3 Kcal/g) calculated in [23].

## Results

### Mycale (Oxymycale) acerata

**Fig 2:**
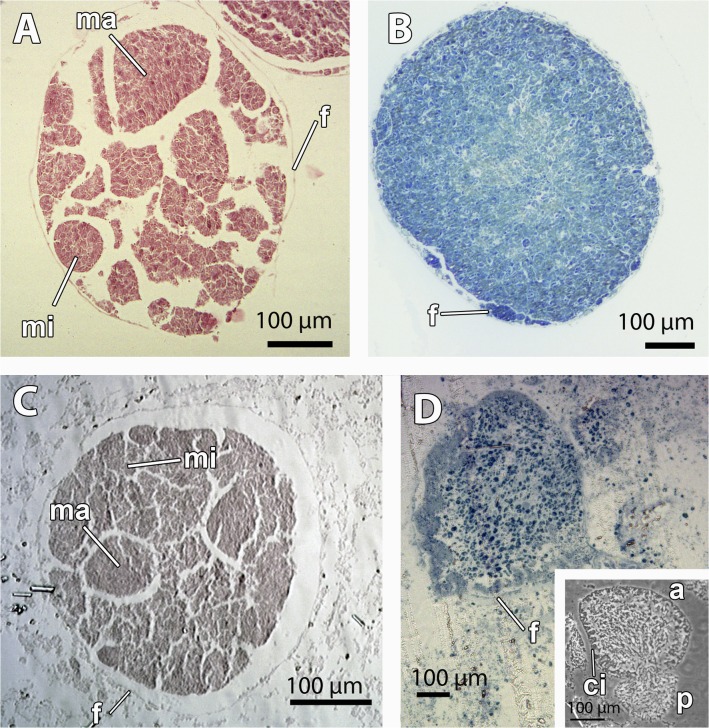
Morphological gross features of the embryos of *Mycale acerata* and embryos and larvae of *Mycale laevis*. A. Mid-stage embryo of *M*. *acerata*, detached from the tissue, showing macromeres (ma), micromeres (mi), and the follicle (f) surrounding the embryo. B. Late-stage embryo of *M*. *acerata*, detached from the tissue. Note the similar size of all blastomeres and the follicle (f) surrounding the embryos. C. Late-stage embryo of *M*. *laevis* within the tissue, showing macromeres (ma), micromeres (mi), and the follicle (f) surrounding the embryo. D. Larva of *M*. *laevis* within the tissue. Note the follicle (f) of the embryo. Inset showing a larva of *M*. *laevis* with large epithelial ciliated cells (ci) oriented in the anterior (a)-posterior (p) axis.

**Fig 3:**
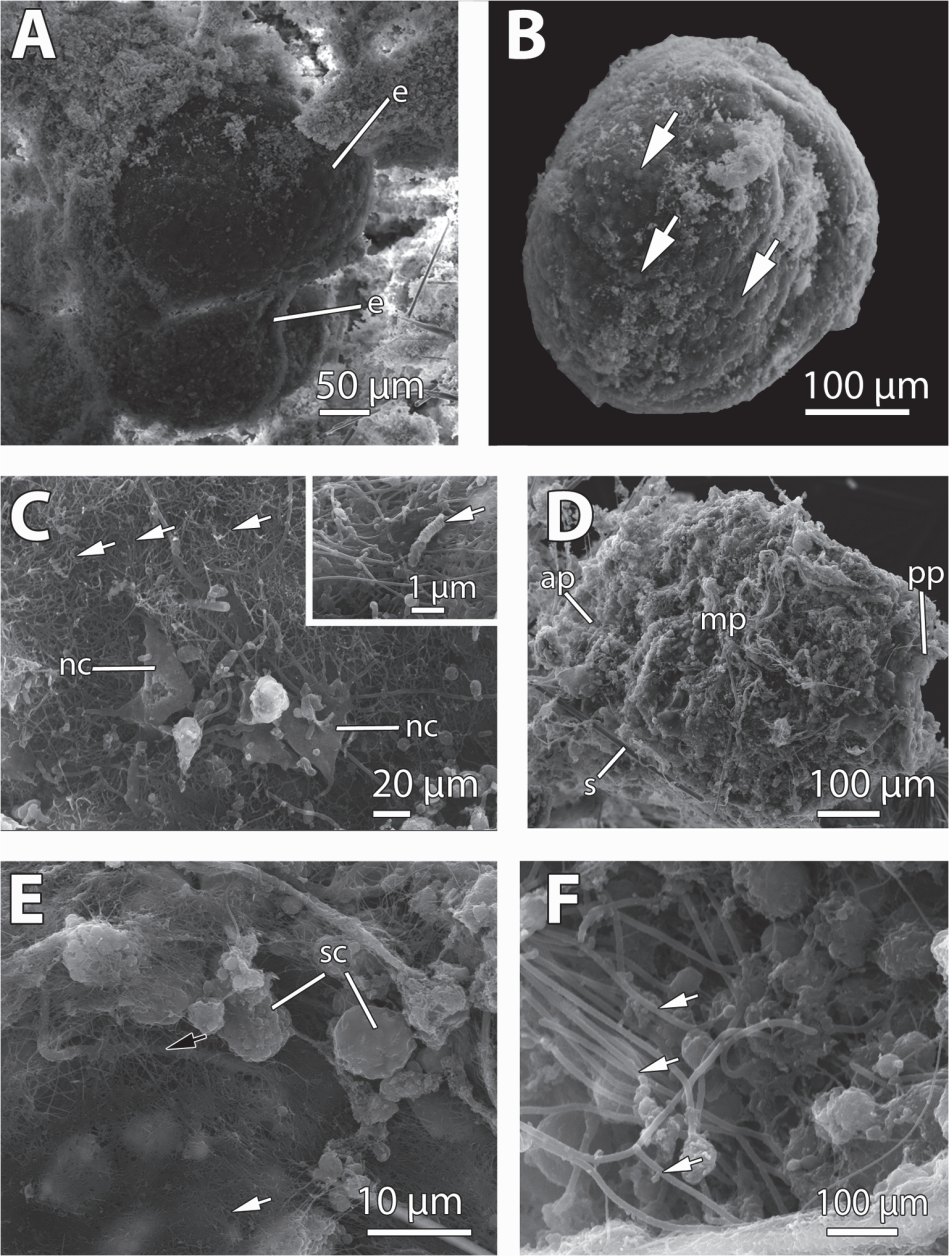
SEM micrographs of embryos of *Mycale acerata* and embryos and larvae of *Mycale laevis*. A. Two mid-stage embryos (e) of *M*. *acerata* brooded within the sponge mesohyl. B. Detached late-stage embryo of *M*. *acerata* showing small micromeres (white arrows). C. Nurse cells (nc) of *M*. *acerata* lying within the collagenous follicle (white arrows). Inset showing rod-shaped bacteria (white arrow) lying on the collagen layer surrounding the embryo of *M*. *acerata*. D. Larva of *M*. *laevis* showing the anterior pole (ap), mid part (mp), and posterior pole (pp). Note the spicules (s) occurring around the larva. E. Posterior bare pole (white arrow) of the larva of *M*. *laevis* surrounded by ciliated cells (black arrow). Note the numerous spherulous cells (sc) in contact with the collagenous layer surrounding the larva. F. Ciliated cells (white arrow) of the mid part of the larva of *M*. *laevis*.

**Fig 4:**
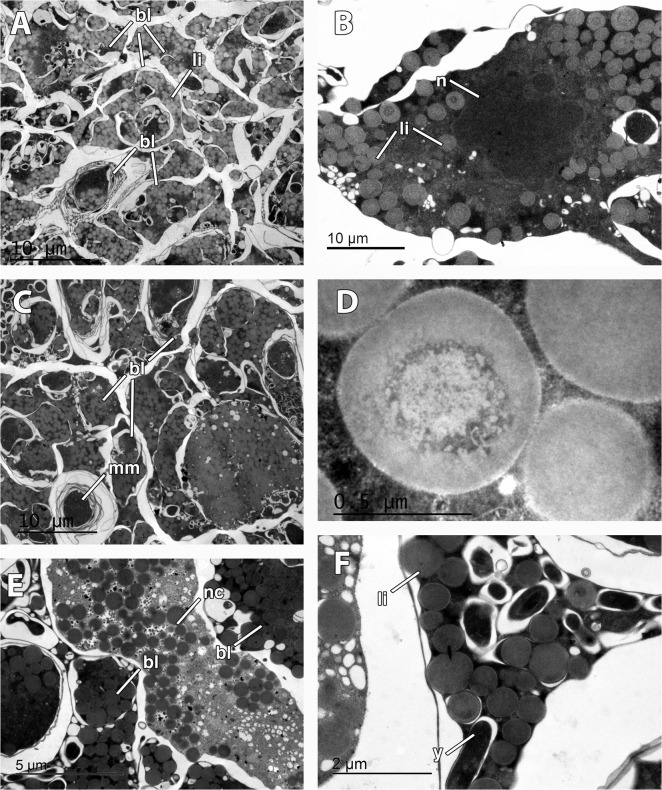
Reproductive elements of *Mycale acerata*. A. Degenerated oocyte in the mesohyl (m) showing lipid droplets (li) and areas with degenerated cytoplasm (dg). B. Cellular (f) and collagen follicle (co) enveloping embryo with round blastomeres (bl) and large lipid inclusions (li). Note the nurse cell (nc) within the lumen of the follicle. C. Nurse cell (nc) in the vicinity of the follicle (f) that contains the embryo (e). Collagen (co) surrounding the follicle. Note the lipid droplets (li) putatively secreted by nurse cells within the embryo. D. Nucleated (n) nurse cell (nc) transporting large lipid inclusions (li) to the embryo blastomeres (bl). Note the collagen (co) layer surrounding the follicle (f). E. Pinacocyte (pi) layer limiting the aquiferous canal (ca) and collagen layer (co) laying underneath. Mesohyl containing symbiotic bacteria (b) and archaeocyte-like cells (a). F. Detail of mesohyl bacteria (b) within collagen (co) accumulations.

The mesohyl of *M*. *acerata* contained mainly two stages of the embryonic development (Figs. 1C, 2A-B, 3A): mid-stage yellow embryos with macromeres and micromeres (Fig. 2A) and late-stage yellow embryos with small blastomeres (Figs. 2B–C, 3B), and also some degenerated vitellogenic oocytes (Fig. 4A). No larvae were found in the individuals studied. Both mid-stage and late-stage embryos were ca. 600 μm in longest diameter (Table 1, Figs. 2A–B, 3A–B), and were surrounded by a follicle composed of a thin collagen layer (1–2 μm) (Figs. 2A–B, 4B–D) and a thin layer of pinacocyte-like cells with relatively few bacteria intermingled with collagen fibers (Fig. 3C and inset). Such follicle was similar to the pinacocyte layer limiting the canals also surrounded by a thick collagen layer of 1–2 μm (Fig. 4E) in which symbiotic rod-like bacteria were lying (Fig. 4E–F). Follicle cells appeared as non-nucleolated flat cells with a nucleus of 5 μm in largest diameter (Figs. 2B, 3C, 4B–D) and a cytoplasm filled with lipid droplets (Fig. 4B–D). Numerous nurse cells (2.5 μm in largest width) were also surrounding the follicle, containing large clusters of lipid droplets (Fig. 4B–D).

**Table 1:** Morphological features of all larvae described in the genus *Mycale*.

Species	Geographic area	Max. diameter (μm)	Ciliation pattern	Color	Spicules	Yolk type	Vertical transmission symbionts	Reference
***Mycale (Aegogropila) contarenii***	Mediterranean, Atlantic	600–800	All—pp	Yellow-brown, colorless pp	pp	lipid/protein	?	[37, 38, 51]
***Mycale (Aegogropila) syrinx***	Mediterranean	650	All—pp	?	pp	?	?	[42, 80]
***Mycale (Carmia) micracanthoxea***	Atlantic	360	All—pp	Yellow, colorless pp	?	?	?	[39]
***Mycale (Carmia) subclavata***	Mediterranean, Atlantic	?	All—pp	?	pp	?	?	[40]
***Mycale (Carmia) fibrexilis***	Atlantic	?	All	Orange, colorless pp	pp	?	?	[41]
***Mycale (Carmia) hentscheli*** (previously *M*. *macilenta)*	Indian Ocean	500	All—pp	Orange-brown, colorless pp	pp	?	?	[45]
***Mycale (Carmia) fistulifera***	Red Sea	450	All—ap & pp	Red	pp	?	?	[47]
***Mycale (Mycale) laevis***	Caribbean Sea	730	Only pp	Green, yellow ring in ap	?	?	?	[50]
	Caribbean Sea	600–700	All—pp	Green	pp	lipid/protein	Yes	present study
***Mycale (Oxymycale) acerata***	Southern Ocean	600*	?	Yellow*	?	?	No	present study
***Mycale* sp.**	?	400	All—ap & pp	White	?	?	?	[49]

Abbreviations: ap = anterior pole, pp = posterior pole

* = only embryos observed

? = unknown, All = totally ciliated;- = except.

**Fig 5:**
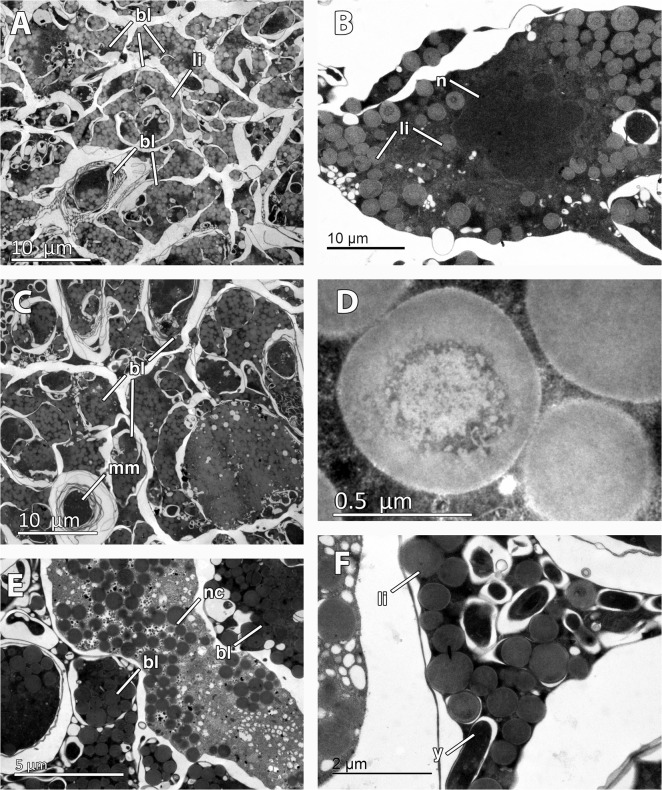
Embryonic features of *Mycale acerata*. A. General view of inner blastomeres (bl) of the embryo, containing abundant lipid droplets (li). B. Detail of nucleated (n) blastomere showing lipid droplets (li). C. Multimembrane inclusions (mm) in the blastomeres (bl) of the embryo. D. Detail of lipid droplet. E. Embryonic cell similar to nurse cell (nc) among the embryo blastomeres (bl). F. Detail of rod-shape inclusions (y) in the blastomeres.

Mid-stage embryos of *M*. *acerata* (Figs. 2A, 3A) were comprised of large macromeres of ca. 200 μm, and smaller micromeres of ca. 50 μm in largest diameter (Fig. 2A) containing numerous lipid droplets (not shown). Late-stage embryos were composed of amorphous intertwined blastomeres (Figs. 2B, 3B, 5A–C) of 12 μm of average maximum diameter (65 μm^2^ in average area) containing large amounts of lipid droplets (Fig. 5A–F) and few electron-dense inclusions (Fig. 5F). Lipid droplets were electron-light non-membrane bound inclusions with an electron-dense core (Fig. 5D). Largest blastomeres in late-stage embryos reached 60 μm in largest diameter (not shown) but most were 12 μm (Fig. 5C). Blastomeres possessed electron-dense cytoplasmic content (Fig. 5B–E) and a non-nucleolated nucleus (Fig. 5B). Several myelin figures were detected scattered in the blastomeres (Fig. 5C). Among the blastomeres, cells with electron-light cytoplasmic content, similar to external nurse cells, were observed (Fig. 5E). No bacteria were detected within the embryo.

In *M*. *acerata*, given that the average radius of cells in the late-stage embryo was 5,2 ± 0,9 μm and the average number of cells in the embryo was 2,51 x 10^05^ ± 1,7 x 10^04^ the amount of yolk contained in the embryos was estimated in 10,42 x 10^06^ ± 1,7 x 10^6^ μm^3^ of yolk, being the lipid yolk the only platelet (Table 2). Approximately 8,23 x 10^−04^ ± 2,8 x 10^−05^ Kcal were contained in each late-stage embryo (given that the density of lipids is ca. 1 mg/ml and its ECF of 7,9 kcal/g; [23]). Assuming that yolk is consumed during larval formation, around 1/3 of the total yolk would remain in the larva (following *M*. *laevis* observations, see below), approximately 2,74 x 10^−04^ ± 4,5 x 10^−05^ Kcal would be the energy contained in the larvae of *M*. *acerata*.

**Table 2:** Yolk content estimations and energetic signatures in the embryos and larvae of *Mycale laevis* and *M*. *acerata*.

	*Mycale laevis*			*Mycale acerata*
	Protein yolk	Lipid yolk	Heterogeneous yolk	Lipid yolk
	platelet	platelet	platelet	platelet
**Average surface (μm** ^**2**^)	1,4 ± 0,2	1,4 ± 0,3	1,6 ± 0.2	0,2 ± 0,1
**Number per cell**	10,6 ± 2,3	1,8 ± 0,2	5,8 ± 1,1	61,3 ± 11,5
**Volume (μm** ^**3**^) **per cell**	3,2 ± 1,02	0,649 ± 0,2	4,29 ± 0,6	38,2 ± 5,9
**Volume (μm** ^**3**^) **per embryo or larva**	893.293,5 ± 228.795,9	95.531,1 ± 43.109,2	1.255.550,75 ± 374.141,5	10.419.922,6 ± 1.709557,4
**Total volume of yolk (μm** ^**3**^) **per embryo or larva**	2,2 x 10^06^ ± 0,5 x 10^06^			10,42 x 10^06^ ± 1,7 x 10^06^
**Energetic signature per embryo or larva** (Kcal)	1,17 x 10^−04^±2,8 x 10^−05^			8,23 x 10^−04^ ±2,8 x 10^−05^

### Mycale (Mycale) laevis

**Fig 6:**
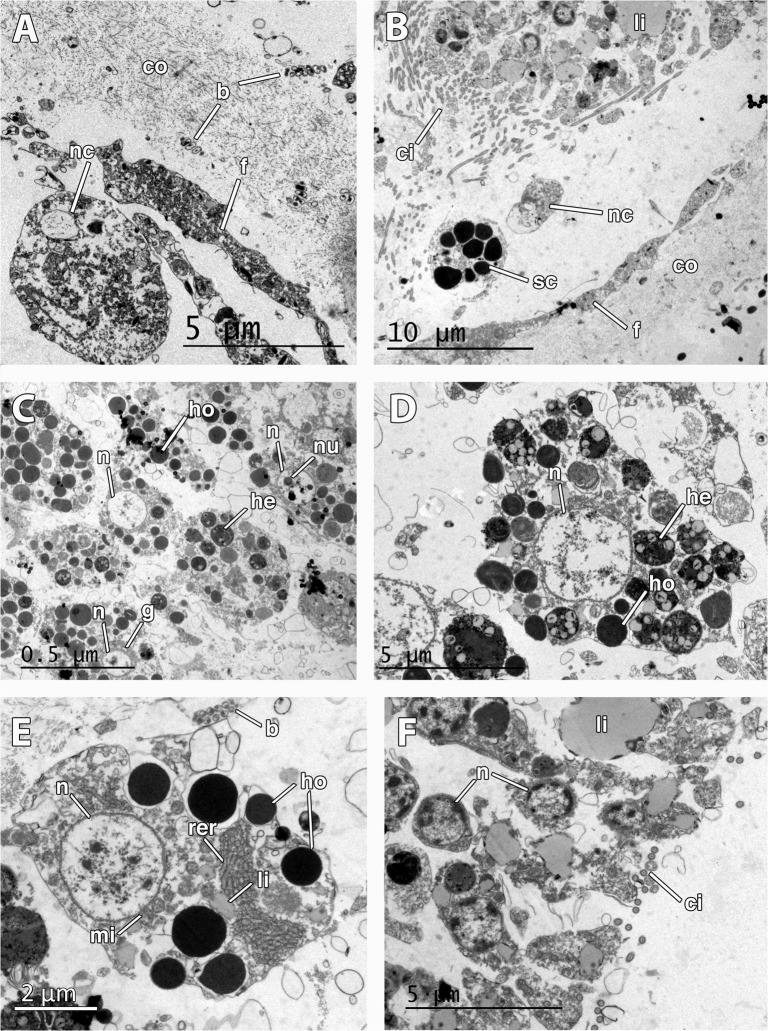
Ultrastructure of the embryo and larva of *Mycale laevis*. A. Cellular follicle (f) and collagen layer (co) surrounding the embryo. Note the presence of a nurse cell (nc) within the lumen of the embryonic follicle and symbiotic bacteria (b) lying in the collagen layer. B. Posterior end of the larva showing ciliated cells (ci) prior to the bare area, and lipid droplets (li) in the cells. Note that nurse (nc) and spherulous cells (sc) have crossed the follicle (f) and are found within the lumen. C. Round nucleolated (nu) cells in the mid part of the larva showing homogeneous (ho) and heterogeneous (he) yolk, and Golgi apparatus (g). Note the round nucleus of the cells (n). D. Close up of nucleated (n) larval cell showing homogeneous (ho) and heterogeneous (he) yolk. E. Detail of nurse cell outside the embryo showing large rough endoplasmic reticulum (rer), non-nucleolated nucleus (n), several mitochondria (mi), and many homogenous yolk platelets (ho). Note the bacteria (b) in the mesohyl. F. Larval ciliated (ci) cells in the posterior end showing large lipid droplets (li).

Two different stages of the embryogenesis were found within the tissue of *M*. *laevis*: late stage embryos and early parenchymella larvae (Fig. 2C–D, 3D). Embryos were round and bluish green in color, 500 μm in longest diameter, while early larvae were also green, bullet-shaped, and slightly larger, ca. 600–700 μm (Table 1, Fig. 1D, 2C–D). Embryos appeared in brooding chambers of 20–30 per chamber (Fig. 1D). Both embryos and larvae were surrounded by a follicle formed by one layer of pinacocyte-like cells (Figs. 2C,D, 6A–B) and a thick layer of collagen of 5 μm (Fig. 3E, 6A–B) with relatively few spirochaete-like bacteria lying on it (Fig. 6A). Follicle cells did not contain lipid inclusions but few protein inclusions and permitted the transit of nurse cells (Fig. 6A–B) and spherulous cells (Fig. 3E, 6B) to the lumen of the embryonic follicle.

**Fig 7:**
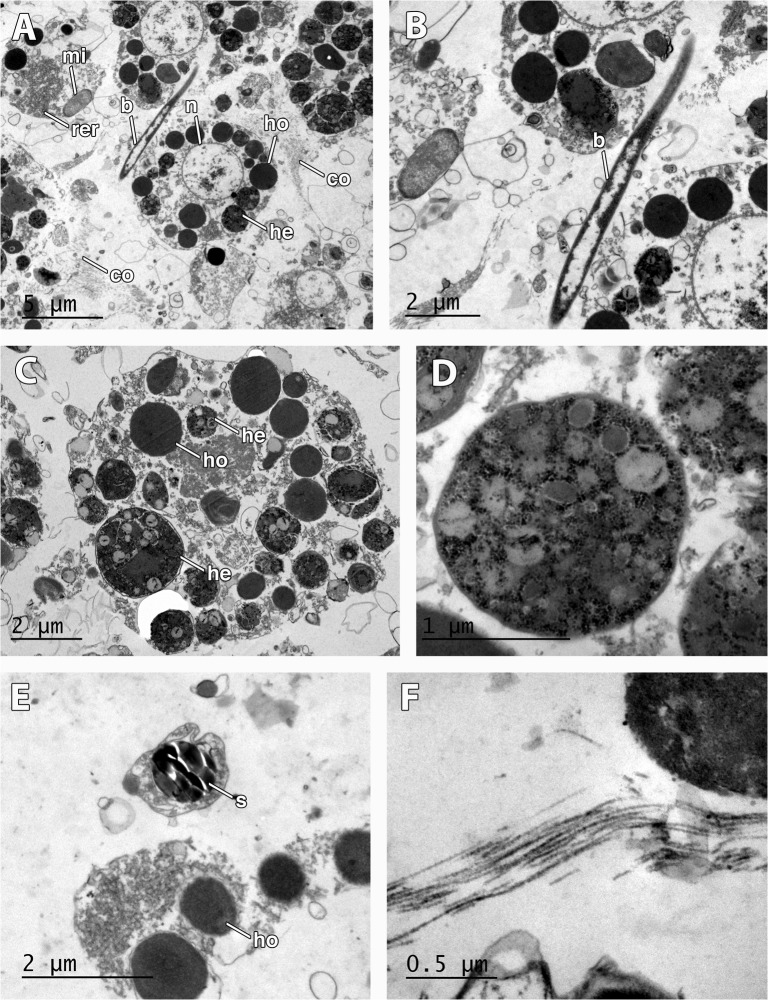
Larval features in *Mycale laevis*. A. Larval cells filled with homogeneous (ho) and heterogeneous (he) yolk, large mitochondria (mi), round large nucleus (n), interspersed bacteria (b), and collagen (co). B. Detail of bacteria (b) among larval cells. C. Detail of morphology of round larval cells with homogeneous (ho) and heterogeneous (he) yolk. D. Detail of heterogeneous yolk platelet. E. Sclerocyte showing a cross-section of siliceous spicule (s) in the mid part of the larva. F. Detail of collagen fibers in the mid part of the larva.

Late-stage embryos were composed of round undifferentiated blastomeres divided into macromeres and micromeres (Fig. 2C). Early larvae (Fig. 3D–F) presented an epithelial layer of elongated ciliated cells (Fig. 3F) of ca. 20 μm of largest diameter and a ring of smaller posterior ciliated cells (Figs. 3E–F, 6F) surrounding a bare posterior pole (Fig. 3E), as well as inner round cells in the mid-part (Figs. 6C–E, 7A–C) with interspersed sclerocytes (Fig. 7E) and collagen (Fig. 7F). Blastomeres in late-stage embryos (Fig. 6C–D) and internal cells in early larvae (Fig. 7A–C) were nucleolated and not ciliated (Figs. 6C–D, 7A–C), and contained numerous heterogeneous and homogeneous yolk platelets, large mitochondria and rough endoplasmic reticulum (Fig. 7B–C). Late-stage embryos and larval internal cells were ca. 9 μm of average largest diameter, and 45,5 μm^2^ in average area. Heterogeneous yolk platelets of both lipid and protein nature (Figs. 6C–D, 7D) were only found in blastomeres and larval cells in anterior and mid parts, and appeared to be membrane-bounded (Fig. 7C). In turn, non-membrane-bounded homogeneous yolk platelets (protein nature) were also found in nurse cells surrounding embryos and larvae (Fig. 6E). Posterior cells of larvae were ciliated and contained large lipid droplets (Fig. 6F). Larval cells contained from half to 1/3 of the yolk contained in the late-stage embryos (approximately 5,6 x 10^06^ ± 1,34 x 10^06^ μm^3^ in embryos and 2,2 x 10^06^ ± 0,5 x 10^06^ μm^3^ in larvae), indicating that yolk was consumed during larval formation. Larval sclerocytes were round cells in cross-section of 1,5 μm that enveloped silica spicules of ca. 1 μm in largest diameter and no inner filament (Fig. 7E). Tylostyles were also observed in the anterior and posterior parts of the larva (not shown). Few collagen fibers found within the medial part of the larvae (Fig. 7A, F). Rod-like bacteria of 5 μm in largest diameter among the cells in the mid-part of the larva (Fig. 7A–B), but not detected in the anterior or posterior parts of the larva.

Given that the average radius of larval cells in *M*. *laevis* was 4,59 ± 0,4 μm and the average number of cells in the larvae was 2,9 x 10^05^ ± 8,1 x 10^04^ the amount of yolk contained in early larvae in *M*. *laevis* was estimated in 2,2 x 10^06^ ± 0,5 x 10^06^ μm^3^ of yolk, being the heterogeneous yolk the dominant platelet (Table 2). Approximately 1,2 x 10^−04^ ± 2,8 x 10^−05^ Kcal were contained in each embryo/larva (given that 1,21 g/ml was the average density of lipoproteins and 4,3 Kcal/g the ECF, and the density of lipids is ca. 1 mg/ml and its ECF of 7,9 kcal/g; [23]).

## Discussion

### Comparative morphology of reproductive elements in *Mycale* species

Both *Mycale acerata* and *M*. *laevis* are brooding species, with several embryonic stages within the same individual, which concurs with the observations in other *Mycale* species [38, 47]. Embryos and larvae of the genus *Mycale* are usually highly pigmented [39, 41, 44, 47], being green or blue in *M*. *laevis* with a lighter posterior pole (this study and [50]) and yellow in *M*. *acerata* (this study). The embryos of *M*. *acerata* and *M*. *laevis* were highly similar in size (ranging from 500 to 750 μm) and shape, being also strongly similar to the embryos and larvae of other *Mycale* species (see Table 1; [38, 39, 42, 45, 46, 47, 49, 50, 51]. Embryos of *M*. *acerata* and embryos and larvae of *M*. *laevis* appeared surrounded by a follicle comprised of collagen (thicker in *M*. *laevis* than in *M*. *acerata*) and a pinacocyte-like cell layer, while in *M*. *fistulifera* also spicules appear surrounding embryos [47].

Most of the larvae of the *Mycale* species studied so far possess spicules exclusively in the posterior part [37, 40, 41, 45, 46, 47]: all spicule types present in adults except for sigmas in *M*. *hentscheli* (previously known as *M*. *macilenta* var. *australis*) [45, 46], tylostyles and sigmas in *M*. *fistulifera* [47], oxytylotes and anisochelae in *M*. *fibrexilis* [41], and subtylostyles, sigmas, toxas, and anisochelae in *M*. *syrinx* [42]. In *M*. *laevis*, we found tylostyles in both the anterior and posterior part of the larvae, and also sclerocytes containing spicules, similar to that found in *Mycale* sp. [49]. Given that no mature larvae were found in *M*. *acerata*, we cannot discard the occurrence of spicules in the larvae of the Antarctic species. Interestingly, it has been suggested that the presence of spicules in larvae may help larval sinking when yolk is depleted and therefore enhance the changes of larvae settlement by contacting the substratum (e.g., [52]).

The larval ciliation pattern in the genus *Mycale* seems to differ slightly from species to species. Among the non-tufted parenchymella larvae described in the different species in the genus, there are examples with entirely ciliated larvae [38], larvae ciliated except for the anterior and posterior part [47, 49], larvae only ciliated in a posterior ring [50], or larvae entirely ciliated except for the posterior pole [37, 39, 40, 42, 45, 53]. In *M*. *laevis*, the ciliation appeared in the entire larva, except for a small bare area in the posterior pole, which contrasts with that observed previously for *M*. *laevis* larvae (orange morph) where ciliation appeared only in a posterior ring [50]. No data on the larval ciliation could be described for *M*. *acerata* since no larvae were found in the samples of this species.

In *M*. *laevis*, spherulous cells appeared in the lumen of the embryonic follicle and close to the collagenous layer of the follicle. In *Mycale* sp. [48, 49], spherulous cells were detected intermingled among the ciliated epithelial cells of the larva. The nature of the spherules in the spherulous cells is unknown in *M*. *laevis* and *Mycale* sp., but given the strong pigmentation of the larvae in the genus *Mycale*, and the observation of pigment granules in the larval epithelium of *M*. *fibrexilis* [41], it could well be that the spherules are pigment accumulations. However, there is an alternative explanation for the presence of spherulous cells in the larvae of *M*. *laevis*. Spherulous cells containing secondary metabolites in *Aplysina fistularis* [54] bear a strong resemblance with the spherulous cells of *Mycale laevis*. Interestingly, unpalatability of larvae of *Mycale laxissima* has been described before related to the use of secondary metabolites against sympatric predators [55]. However, it appears that *M*. *laevis* is not deterrent of sympatric predators [56, 57]; similarly, *M*. *acerata* is non-deterrent against the sympatric predator *Odontaster validus* [58]. Thus, we hypothesize that spherulous cells in *M*. *laevis* might not be involved in defensive strategies but rather in other biological processes.

Only in *M*. *laevis*, bacteria were present, being in the inner part of the larva, suggesting a mechanism of vertical transmission for bacterial symbionts in this species. This is the first time that such a mechanism is reported for any *Mycale* species, a mechanism otherwise very common in other sponges (e.g., [59, 60]).

One of the most striking differences between the embryos of *M*. *laevis* and *M*. *acerata* was the nutrient reserve nature and content. While in *M*. *laevis* the yolk nutrient reserves were comprised of homogeneous protein yolk, lipid droplets, and heterogeneous yolk (mixture of protein, lipid, and glycogen), in *M*. *acerata* embryos appeared to rely completely upon lipid droplets for their further development, survival, and subsequent settlement. Usually, sponges possess different degrees of abundance of protein and lipid yolk and glycogen (e.g., [21, 35, 61, 62]). However, the discovery of embryos entirely containing lipid yolk occurring in *M*. *acerata* is highly remarkable since it is the first time that such a feature is reported for any sponge. However, it is important to note here that protein yolk could perhaps be formed in the latest stage of the embryonic development of *M*. *acerata*, even though this possibility has never been observed in any other sponge before.

### Ecological implications of yolk composition in *Mycale* species

Antarctic marine species face a range of unique environmental challenges like extreme low and stable temperatures, typically between 0 to −1.8°C for most of the year, combined with the most intense seasonality in primary production in the world's oceans and highly seasonal ice scouring [63, 64, 65, 66]. Marine animals are found abundantly at all low temperatures which *per se* do not limit life. There are many different adaptations to enable life in cold ecosystems, like slow growth rates, antifreezing proteins, or psychrophilic enzymes [64, 67]. In relation to reproductive processes, there is a tendency towards long development periods, brooding, producing large eggs, and lecithotrophic strategies, especially in molluscs [64, 68, 69, 70], although there are also many examples of highly abundant marine invertebrates in Antarctica with planktotrophic larvae [69, 71].

Given that the Antarctic ecosystem is highly controlled by its strongly seasonal primary productivity, marine invertebrates have to cope with food scarcity during most of the year [65, 72]. In this sense, one of the most abundant marine invertebrate in Antarctica, the krill *Euphasia superba*, depends entirely on lipid reserves to survive the winter [73, 74]. Also, in Antarctic phytoplankton communities, up to 80% of their fixed carbon is transformed to lipids, compared to the 20% that has been observed in their counterparts of temperate communities [75].

Lipids are the major metabolic energy reserve in the larvae of marine animals [76]. In our study, the carbon stored in yolk reserves in the embryos of the Antarctic *M*. *acerata* was entirely fixed in lipids, while in the Caribbean *M*. *laevis*, lipid yolk was only the 30% of the total nutrient reserves of the embryos and larvae. Fat is an ideal storage material because it liberates twice as much energy as it is liberated by an equal weight of carbohydrate or protein [77]. Fat also serves to buoy floating animals, since it has lower specific gravity than water [77]. In low productivity environments such as the Southern Ocean, larval survival depends upon its energetic content and the metabolic rate at which those reserves are consumed during development [64, 72]. In the Southern Ocean, the importance of low food availability has been raised as the major factor limiting developmental rates in marine invertebrates, being a greater constraint than the influence caused by low temperatures [64, 72]. For instance, during the embryonic development of the sea urchin *Sterechinus neumayeri*, the metabolic rates increase largely during late stages of embryogenesis, when morphogenetic movements occur [78]. Likewise, in fish development, the embryo undergoes intensive cellular movements (gastrulation and epiboly), which require great quantities of energy rich molecules [79]. In the case of *M*. *acerata*, the large storage of lipid droplets could favor rapid morphogenetic movements and therefore quick metamorphosis, as well as a large energetic reserve storage that may enable massive recruitments of larvae. In addition, the large storage of lipids that is estimated to occur in larvae of *M*. *acerata* could reflect a higher buoyancy of the larvae. This higher buoyancy could enable drifting during longer periods in the currents [49] and therefore facilitate dispersal to more distant areas. In summary, large lipid storage in *M*. *acerata* embryonic elements could be behind the remarkable ecological success reported for this species in the Ross Sea and the South Shetland Islands, where these sponges form large and massive populations [28, 29, 30].

This is the first study comparing the embryogenesis in two sponges inhabiting contrasting habitats at an ultrastructural level, which allowed the discovery of divergent strategies in the yolk formation with presumable ecological implications. In particular, the observation of an entirely lipid yolk content in the sponge *M*. *acerata* is unique among the phylum Porifera, and encourages further studies on other Antarctic sponges to establish whether this is a general trend in cold environments.
